# Ultrasound-Assisted Enzymatic Extraction of Polysaccharides from *Tricholoma matsutake*: Optimization, Structural Characterization, and Inhibition of α-Synuclein Aggregation

**DOI:** 10.3390/foods13244150

**Published:** 2024-12-21

**Authors:** Wen Gao, Yang Wang, Fuping Lu, Fufeng Liu

**Affiliations:** Key Laboratory of Industrial Fermentation Microbiology, Ministry of Education, Tianjin Key Laboratory of Industrial Microbiology, College of Biotechnology, Tianjin University of Science and Technology, Tianjin 300457, China; gaowenzb@163.com (W.G.); wangyangswag@163.com (Y.W.); lfp@tust.edu.cn (F.L.)

**Keywords:** *Tricholoma matsutake*, polysaccharides, ultrasound-assisted enzymatic, response surface methodology, antioxidant, Parkinson’s disease

## Abstract

This study optimized ultrasound-assisted enzymatic (UAE) extraction of TMP (*Tricholoma matsutake* polysaccharide) through response surface methodology. The optimal conditions included complex enzyme comprising 1.15% cellulase, 0.60% pectinase, and 0.95% dispase, with ultrasound for 24 min at 84.5 °C and enzyme hydrolysis at pH 5.0. This process yielded 19.74 ± 0.51% TMP, exceeding traditional hot water extraction by over four times. Fourier transform infrared spectroscopy (FT–IR) confirmed that UAE did not alter the structure of TMP. In vitro experiments indicated that TMP-UAE demonstrated enhanced antioxidant properties. Further purification through DEAE-52 and Sephadex G-100 chromatography resulted in a homogenous polysaccharide fraction (TMP). Characterization indicated that TMP has an average molecular weight of 2.79 × 10^4^ Da, composed of fucose, galactose, glucose and mannose in a 2.00:9.44:86.29:2.28 molar ratio. FT–IR indicated the presence of C-O-C glycosidic bonds and pyranyl-type sugar rings. Scanning electron microscopy displayed loose lamellar structures with small pores. Finally, TMP exhibited therapeutic potential against *C. elegans* in Parkinson’s disease, including reducing α-synuclein aggregation, protecting dopaminergic neurons, and prolonging lifespan. This study provides an efficient extraction method for TMP and an insight into its neuroprotective effect in PD *C.elegans.*

## 1. Introduction

*Tricholoma matsutake* is a rare and valuable natural edible and medicinal fungus, also known as the “king of bacteria”. It has the functions of enhancing immunity, as well as anti-virus and anti-cancer properties. Compared with other precious edible and medicinal fungi such as ganoderma, cordyceps, and morchella, *Tricholoma matsutake* ranks first. As early as thousands of years ago, it was already used as a traditional Chinese medicine for the prevention and treatment of diseases in Eastern countries. As early as the Song Dynasty, it was recorded in the “Classification of Materia Medica”. *Tricholoma matsutake* belongs to the genus of *Tricholoma* in the *basidiomycota* phylum, which is mainly found in the coniferous and broad-leaved mixed forests of northeast and southwest China, Japan, Korea, and northern Europe. *Tricholoma matsutake* has received considerable attention for its rare active substances, which include matsutake polysaccharides, matsutake alcohol, and matsutake terpenes. In addition, it also contains proteins, vitamins, lipids, volatile compounds, various active enzymes, and rich dietary fiber.

*Tricholoma matsutake* polysaccharide (TMP) is one of the most important bioactive substances in *Tricholoma matsutake*, which has the characteristics of high research value. Modern studies have shown that TMP has noticeable biological activity, including antitumor [1], antioxidant [2], hypoglycemic [3], immunomodulatory [4], and functional activation effects on macrophages [5]. Nevertheless, due to the scarce sources of TM and the relatively low extraction rate of TMP, it is not enough to meet the growing demand in the pharmaceutical, food and cosmetic fields [6]. Therefore, it is urgent to conduct extensive research to improve its extraction yield and functional activity.

To the best of our knowledge, the most common extraction strategy of polysaccharides is hot water (HW) extraction, as most of the functional polysaccharides are water-soluble polar molecules. However, high extraction temperature and long extraction time may lead to the structural modification of polysaccharides, the degradation of some temperature-sensitive bioactive substances, and the release of a large number of cell wall components and tissue debris, resulting in higher purification costs and lower extraction efficiency [7]. Recently, the UAE method has become a promising choice in the field of plant polysaccharide extraction process optimization, which has the advantages of being fast, efficient, and energy-saving compared with traditional extraction processes [8,9,10]. The UAE method uses papain, cellulase, pectinase, or their composite enzymes formed in a certain ratio to hydrolyze the fiber components of plant cell walls, thus improving the extraction efficiency, which has the characteristics of low energy consumption, high efficiency, and high specificity [11]. In the research by Lin et al., compared to the use of the hot water extraction method, ultrasound-assisted enzyme extraction resulted in higher yield (30.13%) of Shatian pomelo peel polysaccharide [12]. Similar conclusions have also been reported by Mapholi et al. [13].

Recently, plant polysaccharides have attracted extensive attention due to their anti-inflammatory and anti-oxidative and neuroprotective effects in Parkinson’s disease (PD). Gan et al. [14] investigated the neuroprotective effects of Gastrodia elata polysaccharide (GEP), which was administered to 1-methyl-4-phenyl-1,2,3,6-tetrahydropyridine-induced PD mice. Studies have shown that GEP alleviates PD symptoms by inhibiting apoptotic and inflammatory signaling pathways. Tan et al. [15] found that astragalus polysaccharide increased the level of autophagy by activating the PI3K/AKT/mTOR pathway to play a protective role in PD. However, the potential activity of TMP in PD has not been elucidated.

In this study, the UAE method was used to extract TMP, and the extraction conditions were optimized by the response surface method. Subsequently, the characteristics and in vitro antioxidant activity of TMP-HW and TMP-UAE were compared. The structure of purified TMP was characterized In addition, the preventive effect of TMP against PD was evaluated by NL5901 and A30P transgenic *C.elegans*. This study provided an experimental basis for further research on TMP, and has certain guiding significance for the development of TMP food as functional food in PD.

## 2. Materials and Methods

### 2.1. Materials and Reagents

*Tricholoma matsutake* was purchased from the local market in Shangri-La (Yunnan, China). Cellulase (400 U/mg), pectinase (500 U/mg), and dispase (100 U/mg) were provided by Shanghai Yuanye Bio-Technology Co., Ltd. (Shanghai, China). All of the chemicals were of analytical grade unless otherwise specified.

### 2.2. Pretreatment

The sample *Tricholoma matsutake* was dried, crushed with a grinder, and passed through an 80-mesh screen. In total, 95% ethanol absolute (1:20, g/mL) was used to shake the samples for 4.5 h in a constant-temperature shaker at 60 °C (TS-200, Kylin-Bell, Nantong, China), to remove the pigments, lipids, and oligosaccharides. Secondly, centrifugation at 6000 r/min for 20 min (Sigma 3-18KS, Osterode am Harz, Germany) was performed to collect the sediment. After drying in an oven (60 °C) (ZXRD-B5110, Shanghai, China), the sample was passed through a 40-mesh sieve, sealed, and stored in a dark, dry place.

### 2.3. Ultrasonic-Assisted Extraction Process

Pretreated *Tricholoma matsutake* powders were dissolved in ultrapure water (1:30 ratio) and extracted by ultrasound at 180 w (scientz, Ningbo, China). During the ultrasound-assisted extraction process, the temperature was continuously monitored using a digital thermometer (Model, Manufacturer) inserted into the extraction solution. To maintain a constant temperature, a water bath external cooling system was employed to dissipate the heat generated by the ultrasound waves. Adjustments were made manually to ensure that the temperature remained within ±1 °C of the target value throughout the extraction period. After the ultrasonication, the pH was adjusted, and a mixture of 0.6% cellulase, 0.6% pectinase, and 0.6% dispase was added. The solution was digested at 50 °C for 30 min, then centrifuged at 6000 r/min for 20 min to collect the supernatant. The filtrate was concentrated to a quarter of its volume at 60 °C under reduced pressure using a rotary evaporator (RE-210, Zhengzhou, China). Fourfold absolute ethanol was added to the concentrated solution, which was stored at 4 °C. The precipitate was collected by centrifugation, washed, and freeze-dried to yield crude TMP-UAE [16]. Total sugar content was determined by the phenol-sulfuric acid method [17], and crude polysaccharide yield was calculated as follows:

(1) $$\mathrm{Yields}\left(\%\right)=\frac{\mathrm{weight}\;\mathrm{of}\;\mathrm{TMP}}{\mathrm{weight}\;\mathrm{of}\;\mathrm{TM}\;\mathrm{power}}\times 100\%.$$

### 2.4. Optimization of Enzyme-Assisted Extraction

#### 2.4.1. Single-Factor Design for Compound Enzyme-Assisted Extraction of TMP

The single-factor experiments of cellulase addition (0.4%, 0.6%, 0.8%, 1.0%, 1.2%), pectinase addition (0.4%, 0.6%, 0.8%, 1.0%, 1.2%), and dispase (0.4%, 0.6%, 0.8%, 1.0%, 1.2%) addition were carried out according to the single-variable principle. Each experiment varied one factor while keeping the rest constant. It was replicated three times, and the yield of TMP was utilized as the assessment criterion.

#### 2.4.2. Design of Response Experiment for Extraction of TMP by Compound Enzyme

Based on the results, the Box–Bohnken design was used to optimize the extraction process of the complex enzyme and determine the optimal amount of the complex enzyme. The cellulase addition amount (A, 0.8–1.2 mg/mL), pectinase addition amount (B, 0.4–0.8 mg/mL), and dispase addition amount (C, 0.8–1.2 mg/mL) were used as test factors. The yield of TMP was used as the response value.

### 2.5. Optimization of Ultrasound-Assisted Enzymatic Extraction

#### 2.5.1. Design of Single-Factor Experiment for UAE Extraction of TMP

Based on the optimum amount of complex enzyme added, the four single factors—the effect of enzymatic temperature (35, 40, 45, 50, 55, 60 °C), enzymatic pH (3.0, 4.0, 5.0, 6.0, 7.0, 8.0), ultrasonic time (5.0, 10, 15, 20, 25, 30 min), and ultrasonic temperature (40, 50, 60, 70, 80, 90 °C)—on the yield of TMP were further investigated. Only one factor was changed for each experiment and the obtained optimal factor was used as the fixed condition for the next single-factor experiment. Each experiment was repeated three times and the yield of TMP was used as the evaluation standard.

#### 2.5.2. Design of Response Surface Test for UAE Extraction of TMP

Based on the above results, Box–Behnken was used to design a 3-variable, 3-level experiment to optimize the extraction process of TMP. Ultrasound time (A, 20–30 min), ultrasound temperature (B, 70–90 °C), and enzymatic pH (C,4–6) were identified as influencing factors. The design is shown in Table 1.

### 2.6. Hot Water Extraction of TMP

The pretreated *Tricholoma matsutake* powders were dissolved in ultrapure water at the ratio of solid to liquid (1:30, *w*/*v*) and extracted at 90 °C for 1 h. Subsequently, the mixture underwent centrifugation, and the resulting supernatant was harvested. The collected supernatant was then concentrated to 1/5 of its original volume, followed by the addition of four times the volume of absolute ethanol. This mixture was left overnight at 4 °C. Finally, the precipitate was collected by centrifugation at 6000 rpm for 20 min and subsequently lyophilized (ZLGJ-18, Zhengzhou, China) to yield crude TMP.

### 2.7. Antioxidant Activity Assay

The hydroxyl radical scavenging capacity, DPPH scavenging capacity, and ABTS radical scavenging capacity were determined as described, with slight modifications.

#### 2.7.1. Hydroxyl Radical Scavenging Capacity

The hydroxyl radical scavenging was determined using the salicylic acid method [18]. H_2_O_2_/Fe^2+^ generates hydroxyl free radicals through the Fenton reaction, and salicylic acid effectively captures the generated hydroxyl radicals, reacting with them to form a colored product, 2,3-dihydroxybenzoic acid, which exhibits a characteristic absorption peak at 510 nm. When a substance with scavenging ability is added, the color intensity of the product decreases, allowing the hydroxyl radical scavenging capacity of the sample to be determined based on the reduction in absorbance. The reaction system comprises 1 mL of H_2_O_2_ (8.8 mM), 1 mL of FeSO4 (9 mM), 1 mL of salicylic acid-ethanol solution (9 mM), 1 mL of TMP-HW, and TMP-UAEE at varying concentrations (0.625, 0.25, 0.5, 1.0, 1.5, 2.0, and 2.5 mg/mL). H_2_O_2_ was added at the end of the reaction. The reaction was carried out in a water bath (HW SY21-K, Beijing, China) at 37 °C for 30 min. To account for the absorbance of the sample and other solutions, double-distilled water was used as a background instead of hydrogen peroxide, and double-distilled water instead of the sample solution was used as a negative control. The absorbance of the mixture was measured at 510 nm using the full-wavelength scanner (Thermo Fisher Scientific 1500, MA, USA) and calculated as follows:

(2) $$\mathrm{Scavenging}\;\mathrm{effect}\;(\%)=\left[1-(A_{x}-A_{0})/A\right]\times 100,$$

where A_0_ is the absorbance of the blank control solution; Ax is the absorbance after adding the solution; and A is the background absorption value of the negative control solution without sample.

#### 2.7.2. DPPH Scavenging Capacity

DPPH radical scavenging was determined using the assay described by Yin et al. [19]. The DPPH radical, which contains a single electron, is purple in its alcohol solution and exhibits strong absorption at 517 nm. In the presence of antioxidants, DPPH radicals are neutralized, leading to a lighter solution and a reduction in absorbance at 517 nm. The extent of this reduction is directly proportional to the degree of free radical scavenging. Concisely, samples (TMP-HW and TMP-UAEE at concentrations of 0.625, 0.25, 0.5, 1.0, 1.5, 2.0, and 2.5 mg/mL) were mixed with a 2 mM DPPH-ethanol solution in a 1:1 volume ratio and kept protected from light for 30 min. The absorbance at 517 nm was measured using a microplate reader (SpectraMax i3X, Molecular Devices). Deionized water served as the control, and ethanol was used as the blank. The calculation formula utilized was as follows:

(3) $$\mathrm{Scavenging}\;\mathrm{effect}\;\left(\%\right)=\left[\frac{A_{0}-A_{x}}{A_{0}}\right]\times 100,$$

where A_0_ is the absorbance of blank control solution; A_x_ is the absorbance of TMP solution.

#### 2.7.3. Reducing Power Assay

TMP-HW and TMP-UAEE (2 mL) with different concentrations (0.625, 0.25, 0.5, 1.0, 1.5, 2.0 and 2.5 mg/mL) were added to the mixture comprising 2 mL of 1% potassium ferricyanide solution and 2 mL of 0.2 mol/L phosphate buffer (pH 6.6). The mixture was incubated at 50 °C for 20 min, and then 2 mL of 10% trichloroacetic acid (TCA) was added and mixed with rapid shaking. Subsequently, 2 mL of this mixture was reacted with 2 mL of distilled water and 0.4 mL of 0.1% ferric chloride, and the absorbance value at 700 nm was measured after standing for 10 min. [20] The higher the absorbance value, the stronger the reducing power of the sample.

### 2.8. Purification of TMP

The crude TMP-UAE was purified using DEAE-Sepharose Fast Flow (2.6 cm × 20 cm), elution with NaCl solutions (0.1–0.5 mol/L), and deionized water. The flow rate, maintained at 1.0 mL/min, allowed for eluent collection in centrifuge tubes at 10 mL intervals. Then, the primary fraction was advanced to further purification via a Sephadex G-100 column (1.6 cm × 80 cm) with an elution flow rate of 0.2 mL/min (ultra-pure water elution), collecting every 4 mL eluent into individual centrifuge tubes.

### 2.9. Monosaccharide Composition

Monosaccharide composition was analyzed via 1-phenyl-3-methyl-5-pyrazolone (PMP) pre-column derivatization and high-performance liquid chromatography (HPLC) with modifications [21]. Briefly, 5.0 mg of the samples were hydrolyzed with 4.0 mL of 2.0 M trifluoroacetic acid (TFA) in an ampoule at 100 °C for 6 h. Excess TFA was neutralized with NaOH. The hydrolysate of both polysaccharide samples and monosaccharide standards was then reacted with 0.5 M PMP and 0.3 M NaOH at 70 °C for 40 min. Residual alkali was neutralized with 0.3 M HCl, and the solution was extracted three times with chloroform. PMP-labeled samples were analyzed using a 1260 Agilent HPLC system with a Zorbax SB-C18 column (150 × 4.6 mm, 5 µm). The mobile phase consisted of phosphate buffer (0.1 M, pH 6.85) and acetonitrile (82:18, *v*/*v*) at 1.0 mL/min flow rate. Standards included glucose, rhamnose, galactose, mannose, arabinose, and xylose.

### 2.10. Molecular Weight Distribution Analysis

The molecular weight and configuration of TMP were analyzed using high-performance size exclusion chromatography coupled with differential multi-angle laser light scattering and a refractive index detector (HPSEC-MALLS-RI, Thermo, Madison, WI, USA). The system featured a gel exclusion column (Ohpak SB-805 HQ, 300 × 8 mm). The samples were dissolved in a 0.1 M NaNO_3_ aqueous solution, and the mobile phase comprised 0.1 M NaNO_3_ with 0.02% (*w*/*v*) sodium azide, at a flow rate of 0.6 mL/min. Dextrans (Sigma–Aldrich, St. Louis, MO, USA) with different molecular weights (0.5 to 670 kDa) were used as calibration standards.

### 2.11. UV–Vis of TMP

TMP (1 mg) was dissolved with distilled water and prepared into the 2 mg/mL sample solution. The supernatant was completely dissolved by vortex mixing, and the supernatant was centrifuged and scanned by UV–VIS spectrophotometer (1810DASPC, Beijing, China) at 200–400 nm.

### 2.12. Fourier transform infrared spectrometer (FT–IR)

The appropriate amount of dried sample was weighed, mixed, and ground with KBr, and then pressed into tablets. Using an FT–IR (Nicolet iS50, Cambridge, MA, USA), the measurement interval range was controlled in the range of 4000~400 cm^−1^.

### 2.13. Scanning Electron Microscopy (SEM)

An appropriate amount of sample was placed on the microscope slide and fixed with adhesive. After gold spraying, the morphology of the samples was observed by scanning electron microscopy (FEI Apreo, USA). The samples were tested with an accelerating voltage of 2.00 KV.

### 2.14. Caenorhabditis Elegans

#### 2.14.1. Strains and Culture

NL5901 (Punc-54::α-synuclein: YFP + unc-119) and A30P (PCAT-1::α-synuclein:: GFP) *C. elegans* were placed on NGM medium containing *Escherichia coli* strain OP50 and cultured at 20 °C [22]. All *C. elegans* were synchronized to L4 stage by three successive passages and grew well. NL5901 and A30P were transferred to OP50 NGM plates with or without TMP (2 mg/mL), respectively, 50 per plate. Fresh medium was replaced daily for 5 days. *C. elegans* were washed three times with M9 buffer to remove adherent bacteria. Then, 10 worms from each plate were picked and transferred to 2% agar slide, followed by anesthetized with 10 mM levamisole. Finally, the images were observed and taken by laser confocal microscope (Olympus, Japan), and the plaques of α-Syn aggregate larger than 1 μm^2^ were quantified. The fluorescence quantitative analysis was performed using Image J V1.8. software.

#### 2.14.2. Lifespan Assay

NL5901 *C. elegans* at L4 stage were transferred to OP50 NGM plates coated with and without TMP (2 mg/mL). We defined this day as experiment day 0. The living environment of nematodes was changed on time every day to ensure the food was adequate. They were determined to be dead if *C. elegans* did not respond to mechanical stimulation with platinum wire, wandered off the plate, or had their gestational sac truncated. The whole lifespan analysis was repeated at least three times independently.

### 2.15. Statistical Analysis

SPSS 27.0 software was used to analyze the data. All data are expressed as means ± standard deviation (SEM) of three independent replications. One-way analysis of variance (ANOVA) and *t* test were used to determine the differences between groups. *p* < 0.05 was considered statistically significant.

## 3. Results

### 3.1. Optimization of the Optimal Complex Enzyme Addition

**Figure 1:**
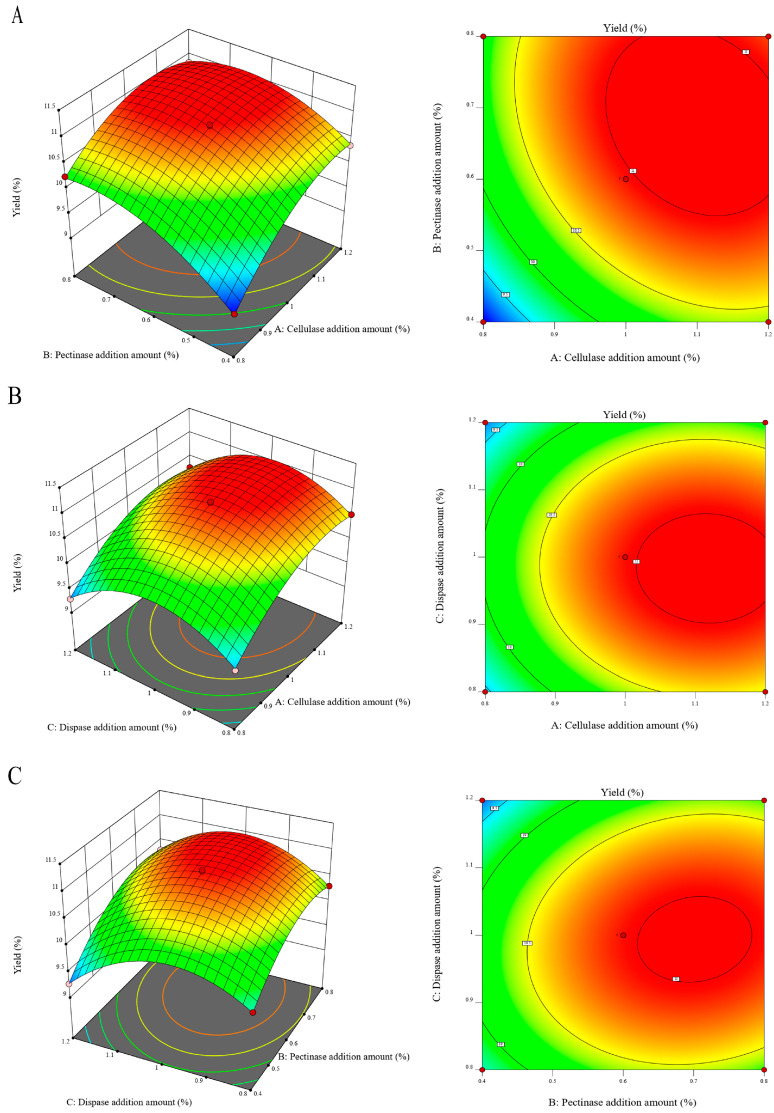
Response surface diagram and contour diagram of the interaction of various experimental factors. (**A**) Cellulase addition amount and pectinase addition amount. (**B**) Cellulase addition amount and dispase addition amount. (**C**) Pectinase addition amount and pectinase addition amount.

The optimal ratio and additional amount of complex enzymes were optimized. The experimental design and results are shown in Tables S1–S3. A 3D response surface was constructed to describe the interaction between factors, with the two independent variables displayed through the 3D surface plot and the other variables fixed at the 0 level. Design Expert (12.0.3.0) software was used to analyze the results. The results are shown in Figure 1. The larger for F-value, the greater the influence of the independent variable on the dependent variable. The 2D contour shape can represent the significance of the interaction between various factors, while the steepness of the 3D response surface effect is positively correlated with its influence on the dependent variable [23]. Combined with Table S4 and Figure S1, the optimal conditions for obtaining the compound enzyme were as follows: the addition amount of cellulase was 1.14%, the addition amount of pectinase was 0.57%, and the dispase was 0.94%. To facilitate the practical operation of the experiment, the amount of complex enzyme was adjusted to 1.15% cellulase, 0.60% pectinase, and 0.95% dispase. Under these conditions, the actual TMP yield was 10.95 ± 0.31% (*n* = 3), which was close to the predicted value (11.03%), as shown in Table S4. The results showed that the response model is suitable for the process of enzymatic extraction.

### 3.2. Optimization of Parameters for Extracting TMP Through UAE

#### 3.2.1. Effect of Enzymolysis Hydrolysis Temperature on the Yield of TMP

**Figure 2:**
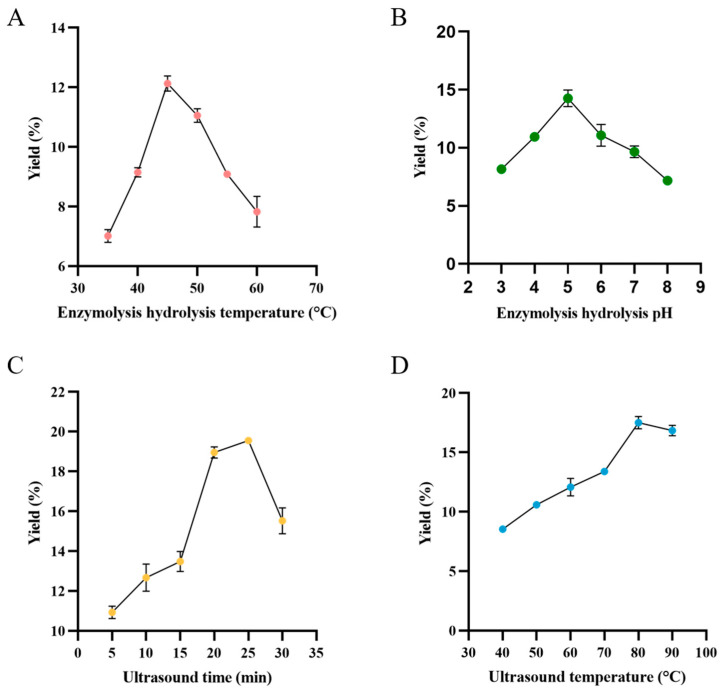
Effects of enzymolysis hydrolysis temperature (**A**), enzymolysis hydrolysis pH (**B**), ultrasound time (**C**) and ultrasound temperature (**D**) on the yield of TMP.

The effect of enzymatic hydrolysis temperature on the yield of TMP was studied from 35 to 60 °C. Figure 2A showed that the yield of TMP rose as the enzymatic temperature increased, peaking at 12.12% when the temperature reached 45 °C. Nevertheless, the yield decreased with the increase in temperature outside of this range. The decrease may be because if the enzymatic hydrolysis temperature is higher than a certain range. Some of the enzyme becomes denatured, leading to inactivation, and the polysaccharide yield is also reduced. Therefore, the optimum enzymatic hydrolysis temperature is 45 °C.

#### 3.2.2. Effect of Enzymolysis Hydrolysis pH on the Yield of TMP

The effect of different pH (3, 4, 5, 6, 7, 8) on TMP extraction efficiency was investigated when other extraction factors were fixed. Figure 2B showed that the yield of TMP increased steadily with the increase in pH from 3 to 5, reaching a maximum of 14.27% at pH 5 [24]. However, beyond pH 5, TMP production began to decrease, indicating that the activity of the enzyme complex was impaired at higher pH levels. This observation suggested that we need to perform subsequent experiments in the pH range of 4 to 6 determined in this study to ensure optimal TMP extraction efficiency.

#### 3.2.3. Effect of Ultrasound Time on the Yield of TMP

The effect of sonication time (5–30 min) on the yield of TMP was studied. TMP yield increased from 5 to 25 min, peaking at 19.49% at 25 min, before showing a decline (Figure 2C). Specifically, the yield significantly improved between 15 and 20 min, indicating that ultrasonic treatment effectively accelerated the TMP release and extraction. However, the decrease in yield beyond 25 min indicated that prolonged exposure can lead to undesirable effects, such as damage to the polysaccharides due to the high system pressure and shear forces, as well as potential alterations in enzyme activity due to temperature changes [25]. These findings highlighted the need to strike a balance between enhancing mass transfer and minimizing detrimental impacts on the TMP. Thus, this study identified 25 min as the optimal ultrasound duration.

#### 3.2.4. Effect of Ultrasound Temperature on the Yield of TMP

To study the effect of ultrasonic temperature on the yield of TMP, we utilized temperatures ranging from 40 °C to 90 °C for extraction.

The results indicated a positive correlation between temperature and TMP yield from 40 to 80 °C, after which the yield began to decline smoothly (Figure 2D). This result can be attributed to cavitation and diffusion effects, both of which are enhanced by increasing the temperature. Cavitation promotes the diffusion of polysaccharide substances and improves the extraction yield of polysaccharides. However, beyond 80 °C, the reduction in surface tension and viscosity inhibits ultrasound activity, ultimately resulting in decreased TMP yield [26]. Consequently, 80 °C was identified as the optimal temperature for our experiments.

### 3.3. Design of Response Surface Test for Extraction of TMP by Ultrasonic Complex Enzyme Method

Based on the single-factor experiment, the extraction process of TMP was optimized. The specific experimental design and test results are shown in Table 1.

Design Expert (12.0.3.0) software was used to conduct multiple regression fitting analysis of the experimental results, and the second-order multinomial regression Equation (4) of the yield of TMP (Y) on the independent variables of ultrasound time (A), ultrasound temperature (B), and enzymatic hydrolysis pH (C) of the complex enzyme was obtained:

Y = 19.56 − 11.27A + 11.58 B − 0.6178C− 0.4077AB − 0.44281 AC + 0.7729 BC − 2.9A^2^ − 2.82 B^2^ − 4.09 C^2^. (4)

The analysis of variance was performed on the multiple regression model. *p*-values served as a statistical measure to assess the significance of each coefficient. A smaller *p*-value denoted a greater significance for the respective coefficient [27]. As shown in Table 2 and Table 3, the F value reached 315.88, with a *p*-value less than 0.0001, indicating substantial statistical significance for the regression model. The linear coefficients (A, B, C), quadratic coefficients (AB, AB, BC), and cross-product coefficients (A^2^, B^2^, C^2^) presented extreme significance (*p* < 0.01), suggesting that they all significantly impacted the yield of TMP. Based on the analysis of variance, the influence ranking of each factor on TMP yield is as follows: ultrasonic temperature (B) > ultrasonic time (A) > enzymatic hydrolysis pH (C). The *p* value of the misfitting term was 0.4627 > 0.05, and the *p*-value of the Lack of Fit was not significant (*p* > 0.05), indicating that the regression equation was suitable for the prediction model. In addition, R^2^ reflects the proportion of change in the model itself rather than random error. R^2^ = 0.9986 and Adjusted R^2^ = 0.9968 showed that the actual value fitted well with the predicted value. The coefficient of variation (C.V.) was 11.32% indicating that the experimental results were reliable and accurate.

### 3.4. Analysis of Response Surfaces

**Figure 3:**
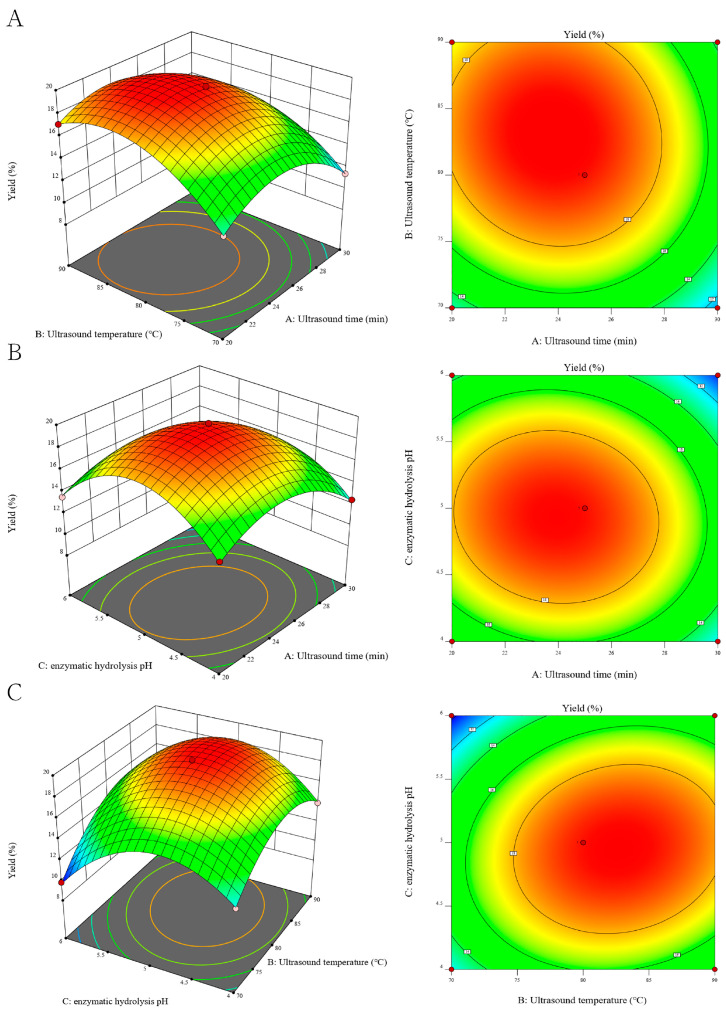
Response surface diagram and contour diagram of the interaction of ultrasonic time (**A**), ultrasonic temperature (**B**) and complex enzyme hydrolysis pH (**C**).

To describe the interaction between the factors, a 3D response surface was constructed with the two independent variables displayed through the 3D surface plot, while the other variables were held constant at zero level. The response surface and contour plots can visually reflect the influence degree of interaction on response values. The steeper surface and denser contour lines indicate more significant influence, with contour lines closer to the ellipses signifying stronger interaction [28]. The greater the influence of each factor, the steeper the slope of the response surface [29]. Figure 3A showed that the highest TMP yield was achieved when the ultrasound temperature increased from 70 to 83 degrees, and ultrasound time increased from 20 to 24 min. Beyond this range, the TMP yield gradually decreased. Similarly, the impact of other variables on TMP yield can be analyzed separately (Figure 3B,C). The interaction between ultrasound temperature and enzyme pH had the most substantial effect on TMP yield, while the interaction between ultrasound time and temperature had minimal impact. These findings align with the results of previous ANOVA analyses.

These results further suggest that the ultrasound-assisted enzymatic method can improve TMP extraction yield. On this basis, the optimal process conditions were obtained: the ultrasonic time was 23.455 min, the ultrasonic temperature was 84.475 °C, and the pH of complex enzyme hydrolysis was 5.012. Under these parameters, the maximum predicted TMP extraction rate was 19.872%. For the convenience of practical operation, the experimental verification was carried out under the parameters of ultrasonic temperature of 84.5 °C, ultrasonic time of 24 min and complex enzyme hydrolysis pH of 5. Table 4 shows that under these parameters, the extraction yield of TMP was 19.74 ± 0.51% (*n* = 3), which was close to the theoretical predicted value. These results indicated the effectiveness of the response model in optimizing UAE extraction processes.

### 3.5. Effects of Different Extraction Methods on TMP

#### 3.5.1. Extraction Efficiency

The effect of different extraction methods was evaluated by comparing the TMP yield, polysaccharide content and protein content. As shown in Table 5, under identical solid–liquid ratio conditions, the TMP extraction yield varied by the method: UAE significantly outperformed HW. Specifically, the yield of TMP from the UAE method reached 65.02%, while that from the HW method was only 37.39%. Notably, ultrasound effectively disrupted cell walls, thereby facilitating the release of polysaccharides, while treatment with complex enzymes (cellulase, pectinase, and dispase) also contributed to cell wall degradation and promoted polysaccharide exudation. These results indicated that UAE extraction not only enhanced TMP yield but also improved its sugar content.

Enzymatic deproteinization, which hydrolyzes proteins within polysaccharides by proteases, provides a milder and safer method. However, it does not achieve complete protein removal [30]. The protein content in TMP extracted by UAE surpassed that of TMP extracted with HW. This may be due to the addition of complex enzymes in the UAE method, which exhibits hydrolytic activity and is dissolved during extraction, resulting in more protein component incorporation [31]. Thus, despite the deproteinization step, the TMP extracted by the UAE method still contains more protein impurities.

#### 3.5.2. IR Analysis

**Figure 4:**
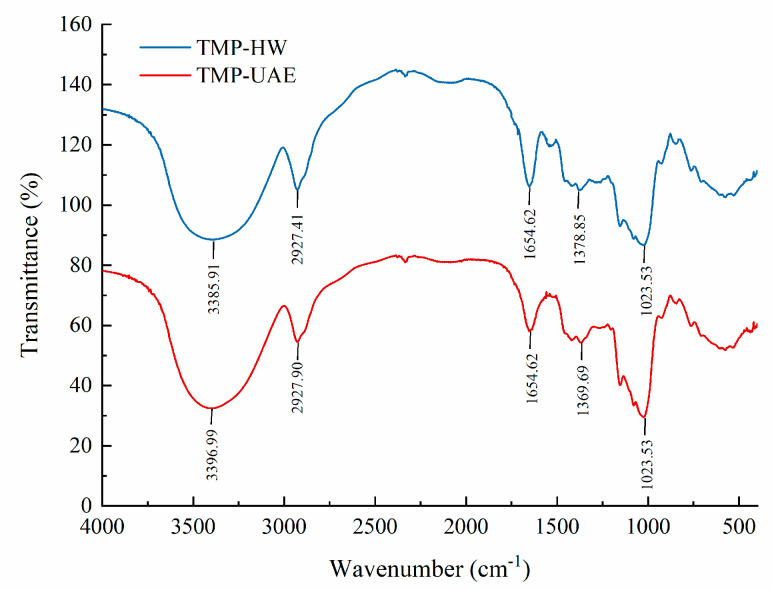
The FT–IR spectra of TMP-HW and TMP-UAE.

Different extraction methods’ influence on the TMP structure was examined based on the responsiveness of FT-IR spectra to organic functional groups. As shown in Figure 4, both TMP-HW and TMP-UAE exhibited a broad and strong characteristic peak near 3400 cm^−1^, indicating the presence of O-H stretching vibration [32]. Additionally, the absorption peaks at 2927.41 cm^−1^ and 2927.90 cm^−1^ indicated the presence of asymmetric bending vibration of C-H bonds, which belong to the characteristic peaks of polysaccharides [33]. Spectral peaks at 1654.62 cm^−1^ were attributed to C-O stretching vibration in the carbonyl group [34]. The absorption peaks at 1378.85 cm^−1^ (TMP-UAE) and 1369.69 cm^−1^ (TMP-HW) represented C-H vibrations [35]. Furthermore, absorption peaks in the range of 1200–1000 cm^−1^ indicated the presence of pyranose structures [36]. These results suggested that the TMP extracted by UAE and HW had similar typical absorption peaks, indicating that UAE had no significant effect on the main chemical structure of polysaccharides and was an efficient and reliable extraction process.

#### 3.5.3. SEM Analyses

**Figure 5:**
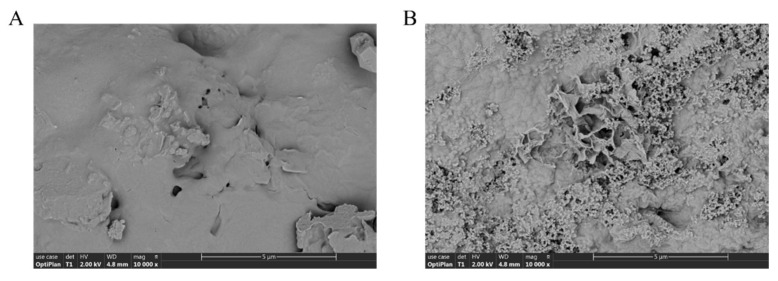
SEM images of TMP-HW (**A**) and TMP-UAE (**B**) (10,000×).

Scanning electron microscopy (SEM) was used to analyze the surface structure of TMP prepared by the two methods. As shown in Figure 5A, TMP-HW has a compact structure with a smooth and has no obvious pores. This observation is consistent with the proposed mechanism that conventional hydrothermal treatment may promote the formation of more homogeneous and compact polysaccharide structures. In contrast, TMP-UAE has a flaky structure with an uneven surface and different bubble pore sizes (Figure 5B). This difference in morphology can be attributed to the effect of the ultrasound-assisted enzymatic method, which induced the formation of voids and pores in the polysaccharide structure [37].

### 3.6. Antioxidant Activity of TMP

In molecular biology, high levels of free radicals have been closely linked to the onset of degenerative processes. This increase can heighten oxidative stress, resulting in inadequate cell function, aging, and potentially diseases [38]. Hence, evaluating the antioxidant capacity of polysaccharides becomes essential for assessing their biological efficacy.

**Figure 6:**
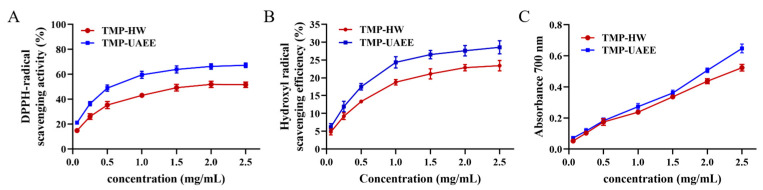
In vitro antioxidant activity of TMP obtained by different extraction methods. (**A**) DPPH radical scavenging activity; (**B**) hydroxyl radical scavenging activity; (**C**) ABTS radical scavenging activity. The concentrations of TMP-HW and TMP-UAEE were 0.625, 0.25, 0.5, 1.0, 1.5, 2.0 and 2.5 mg/mL. The data are presented as mean ± SD (*n* = 3).

Activity DPPH, a stable free radical, can accept an electron or hydrogen radical to become a stable diamagnetic molecule and decrease the absorbance at 517 nm, which has been widely used to investigate radical scavenging activity of natural polysaccharides [39]. As shown in Figure 6A, at lower concentrations (0–1.5 mg/mL), both TMP-UA and TMP-UAEE exhibited a dose-dependent effect on the scavenging of DPPH free radicals. Furthermore, TMP-UAE exhibited enhanced efficacy in DPPH radical scavenging at equivalent concentrations compared to TMP-HW. Notably, the concentration of TMP-HW required to achieve a 50% DPPH scavenging rate was 1.79 mg/mL, while that of TMP-UAE was 0.60 mg/mL.

Hydroxyl radical is the most harmful free radical to living organisms [40]. This free radical oxidizes and damages proteins, nucleic acids, sugars, and other substances, leading to cell necrosis or mutation [41]. As shown in Figure 6B, the scavenging rate of hydroxyl radicals exhibited a positive correlation with the increasing concentrations of TMP-HW and TMP-UAE. However, the scavenging rate remained below 30% even at a concentration of 2.5 mg/mL. Furthermore, the IC50 for hydroxyl radical scavenging activity of TMP-UAE and TMP-HW were determined to be 0.63 mg/mL and 0.50 mg/mL, respectively.

The antioxidant activity of natural polysaccharides correlated with their reducing components, capable of reducing potassium ferricyanide. The Fe^3+^-potassium ferricyanide complex showed peak absorbance at 700 nm. Evaluating reducing power is essential for assessing the antioxidant capacity of natural polysaccharides. Figure 6C shows that the reducing power of TMP-HW and TMP-UAE increased with concentration, with negligible differences from 0 to 2.0 mg/mL. At the absorbance of 0.5 for reducing power, the effective concentrations of TMP-UAEE and TMP-HW were 1.96 mg/mL and 2.35 mg/mL, respectively. To summarize, TMP-UAE exhibited comparatively high antioxidant activity.

### 3.7. Structural Characterization of TMP

#### 3.7.1. Monosaccharide Composition and Molecular Weight Analysis

**Figure 7:**
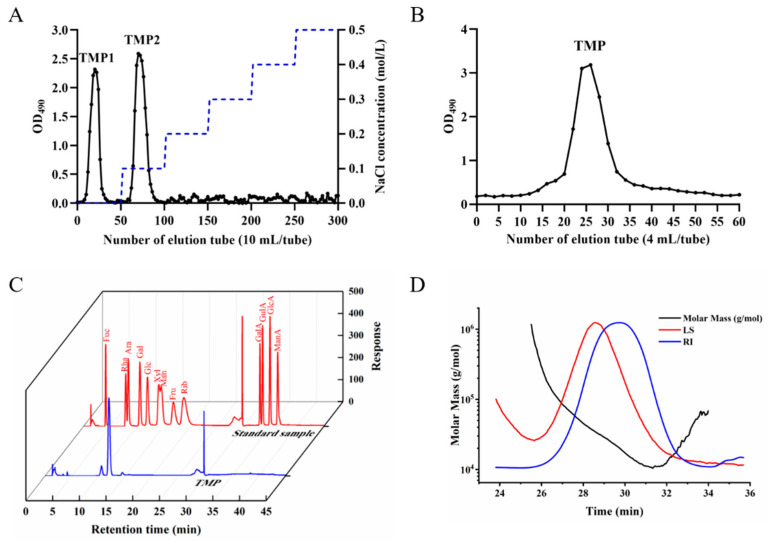
Isolation and purification of TMP. (**A**) The elution curve of TMP1 and TMP 2 on the DEAE-52 chromatography column. (**B**) Elution curve of TMP on Sephadex G-100 chromatography column. (**C**) TMP monosaccharide composition. (**D**) TMP average molecular weight.

The polysaccharide content in each tube was measured by phenol-sulfuric acid method and the elution curves were generated. Two fractions of TMP-1 and TMP-2 were obtained by elution with a DEAE column (Figure 7A), and then a single symmetric elution peak was obtained by elution with Sephadex G-100 (Figure 7B). The eluent of the same peak centrifuge tube was collected, concentrated, and cryoprecipitated to obtain the purified polysaccharide component TMP. The polysaccharide content was 86.39 ± 4.557%.

The monosaccharide composition of TMP was determined based on the retention times and standard curves related to monosaccharide standards. Figure 7C revealed that TMP was mainly composed of fucose (Fuc), galactose (Gal), glucose (Glc), and mannose (Man), with molar percentages of 2.00:9.44:86.29:2.28. These data indicated that Glc serves as the primary component of the TMP sugar chain framework. Additionally, the molecular weight and chain conformation of TMP were characterized by a single symmetric peak (Figure 7D), suggesting that TMP is a relatively homogeneous polysaccharide. The average molecular weight (Mw) of TMP was 27.922 kDa. Previous research utilized the monosaccharide composition and molecular weight of polysaccharides as indicators, employing principal component analysis and cluster analysis to categorize polysaccharides with antioxidant activity into two groups. The first group accounted for 80.8% and had a molecular weight of approximately 28 kDa, predominantly composed of Glc. The second group represented 19.2% with a molecular weight of around 186 kDa, primarily consisting of Ara. These findings suggested a potential correlation between the molecular weight and monosaccharide composition of polysaccharides and their antioxidant activity [42]. Furthermore, Wang et al. indicated that low molecular weight polysaccharides are more susceptible to reactive free radicals and oxidants [43]. These results support the notion that TMP exhibits antioxidant properties.

#### 3.7.2. Ultraviolet-Visible Light Analysis and Morphological Structure Observation of TMP

**Figure 8:**
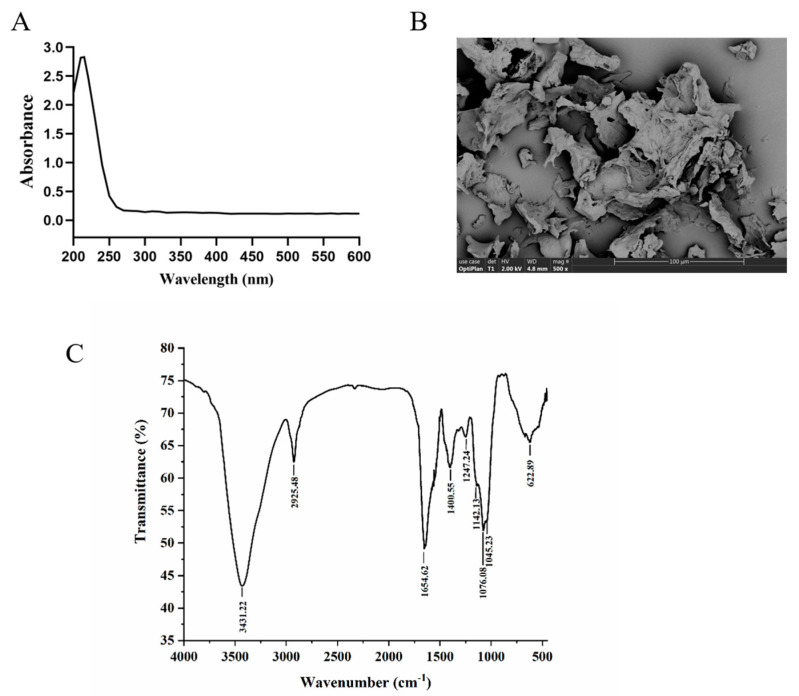
UV–visible spectra, scanning electron microscope and FT-IR spectra of TMP. (**A**) Ultraviolet-visible light analysis. (**B**) SEM images of TMP. (**C**) The FT–IR spectrum of TMP.

The UV scanning results of TMP are shown in Figure 8A, and there is no UV absorption peak at 260 nm and 280 nm wavelength, indicating that TMP does not contain proteins and nucleic acids. The SEM image of TMP is shown in Figure 8B. TMP presents loose lamellar structures clustered together with small pores of different sizes on its surface. It is reported that ultrasound-assisted extraction of polysaccharides from *Flammulina velutipes* exhibited a similar microstructure [44]. Furthermore, research indicates that porous and lamellar conformations can fully expose the active sites of polysaccharides and increase their specific surface area, which facilitates the utilization and absorption of polysaccharides, ultimately enhancing their antioxidant activity [42].

#### 3.7.3. FT–IR Analysis

The FT–IR spectrum of TMP is shown in Figure 8C, displaying absorption peaks ranging from 500 to 4000 cm^−1^. A significant absorption peak at 3431 cm^−1^ indicated O-H stretching vibrations. The peak at 2925 cm^−1^ suggested that TMP possesses structural features characteristic of polysaccharides. Absorption peaks at 1654 cm^−1^ and 1400 cm^−1^ align with the asymmetric and symmetric stretching vibrations of carboxyl groups, respectively. The absence of an absorption peak near 1540 cm^−1^ confirms the lack of N-H groups in TMP, aligning with its UV spectral analysis. Furthermore, three absorption peaks between 1000 and 1200 cm^−1^ indicated the presence of C-O-C glycosidic bonds and pyranyl-type sugar rings, with an absorption peak at 622 cm^−1^ further supporting the existence of pyranyl-type sugar rings.

### 3.8. The Protective Effect of TMP Against C. elegans in Parkinson’s Disease

The α-synuclein is a synaptic protein that is widely distributed throughout the central nervous system and plays a crucial role in maintaining synaptic function under normal conditions. However, the abnormal aggregation of α-synuclein can induce damage to dopaminergic neurons, leading to the onset of PD [45]. The generation of oxidative stress in the brain increases ROS, which promotes the formation of α-synuclein oligomers. Research indicates that some polysaccharides with antioxidant properties can prevent the oxidative modification of α-synuclein by scavenging ROS, thereby preserving its normal structure and function [14,46,47]. Furthermore, the presence of hydroxyl and carboxyl groups in the polysaccharide structure may facilitate polar interactions with amino acid residues on the surface of α-synuclein, thus maintaining the stability of its structure and inhibiting α-synuclein aggregation. Therefore, the inhibitory effect of TMP on α-synuclein aggregation and te protection of dopaminergic neurons in *C. elegans* was investigated [48]

**Figure 9:**
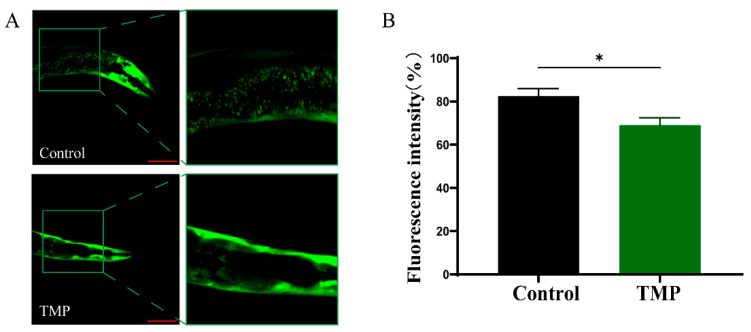
TMP inhibited α-Syn aggregation in NL5901 *C. elegans*. (**A**) Microscopic image of NL5901 *C. elegans* after 5 days of treatment with and without TMP. (**B**) The relative fluorescence intensity of α-synuclein aggregation plaques quantified using Image J. Scale bar is 100 µm. The data are expressed as mean ± SD. *p* < 0.05 was statistically significant (* *p* < 0.05 compared to the Control group).

NL5901 *C. elegans*, which expresses human α-synuclein fused with green fluorescent protein, facilitated the direct observation of α-synuclein aggregation state [49]. Figure 9A,B demonstrated that NL5901 *C. elegans* exhibited significant fluorescence α-synuclein aggregation after 5 days, which markedly reduced upon TMP treatment, showing a 16.29% decrease in fluorescence intensity.

**Figure 10:**
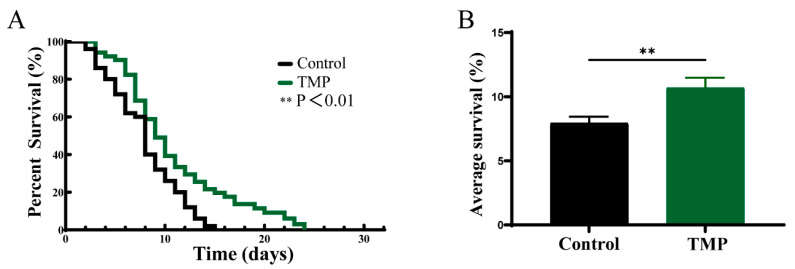
TMP extended the lifespan of NL5901 *C. elegans*. (**A**) Survival curves of NL5901 strains of *C. elegans* treated with and without TMP. (*n* = 50 for each group). (**B**) Comparison of the average lifespan of NL5901 *C. elegans* in the presence and absence of TMP. The mean survival day was assessed using the Kaplan–Meier method, and the log-rank test determined statistical significance to calculate the *p* value. The data are expressed as mean ± SD. *p* < 0.05 was statistically significant (** *p* < 0.01 compared to the Control group).

Moreover, α-synuclein aggregation can induce neurotoxicity, intracellular metabolic abnormalities, and inflammatory responses, affecting lifespan [50]. In our experiments, compared to the untreated group, TMP treatment decreased the mortality rate of NL5901 *C. elegans*, extending their lifespan to approximately 24 days (Figure 10 and Table 6). The average lifespan of TMP-treated NL5901 *C. elegans* was 10.7 days, representing a 34.76% increase over the control group (7.94 d).

**Figure 11:**
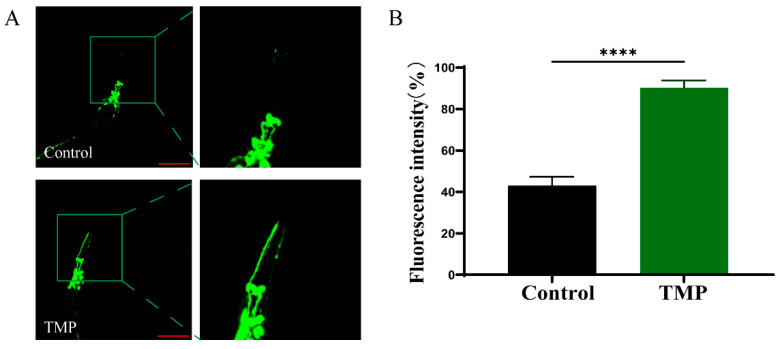
TMP-protected dopaminergic neurons of A30P *C. elegans*. (**A**) Microscopic image of A30P *C. elegans* after 5 days of treatment with and without TMP. (**B**) The relative fluorescence intensity of DAergic Neurons quantified using Image J. Scale bar is 100 µm. The data are expressed as mean ± SD. *p* < 0.05 was statistically significant (**** *p* < 0.0001 compared to the Control group).

The α-synuclein aggregation is an important pathogenic cause of dopaminergic neuron degeneration. The A30P strain expresses human α-synuclein, facilitating the identification of dopamine neuron damage from α-synuclein aggregation, detectable via green fluorescence in eight dopamine neurons and their axonal projections. The dendritic arbors of the A30P *C. elegans* gradually diminish during growth and development, with a 90% reduction in CEP expression [51]. Compared with the untreated group, the average fluorescence intensity of CFP dendrites in GFP-labeled DA neurons in the TMP treatment group was significantly increased (Figure 11).

The collective findings indicated that TMP can protect dopaminergic neurons from degeneration and extend lifespan by inhibiting the α-synuclein aggregation.

## 4. Conclusions

In this study, ultrasound-assisted enzymatic extraction of TMP was optimized, resulting in a yield of 19.74 ± 0.51% using a complex enzyme mixture of 1.15% cellulase, 0.60% pectinase, and 0.95% dispase under ultrasonic conditions of 24 min, 84.5 °C, and pH 5.0. This yield was over four times greater than that achieved with the HW extraction method (4.36%). FT–IR and SEM analyses indicated that the UAE method did not significantly alter the main structure of TMP. In vitro antioxidant tests demonstrated that TMP-UAE had strong antioxidant properties. Further purification by DEAE-52 and Sephadex G-100 chromatography produced a homogeneous polysaccharide fraction of TMP, with an average molecular weight of 2.79 × 10^4^ Da, composed of fucose (Fuc), galactose (Gal), glucose (Glc), and mannose (Man) in a molar ratio of 2.00:9.44:86.29:2.28. TMP features C-O-C glycosidic bonds and pyranyl-type sugar rings, with a loose lamellar structure and surface clusters of small pores, as shown by scanning electron microscopy. Furthermore, TMP demonstrated notable effects in transgenic *C. elegans* models, including the inhibition of α-synuclein aggregation in NL5901 *C. elegans* and protection against dopaminergic neuronal damage in A30P *C. elegans*, which subsequently led to an extension of lifespan in NL5901 *C. elegans*. In conclusion, the UAE method substantially enhanced TMP extraction yields, highlighting its potential for neuroprotective effects in *C.elegans.*

## Figures and Tables

**Table 1:** Box–Behnken response surface design and the corresponding response values.

Run	Ultrasound Time (Min)	Ultrasound Temperature (°C)	Enzymatic pH (%)	Yield (%)
1	25	80	5	19.5222
2	25	70	4	12.3672
3	30	90	5	13.7723
4	25	80	5	19.5244
5	20	70	5	13.0967
6	30	70	5	11.3994
7	30	80	4	12.4545
8	25	70	6	9.82527
9	30	80	6	10.1235
10	25	80	5	19.7762
11	25	90	4	13.9369
12	25	80	5	19.7066
13	25	80	5	19.2754
14	25	90	6	14.4867
15	20	80	6	13.5575
16	20	80	4	14.1764
17	20	90	5	17.1004

**Table 2:** Regression model analysis of variance.

Source	Sum of Squares	df	Mean Square	F-Value	*p*-Value	
Model	7.24	9	0.8044	315.88	<0.0001	significant
A-Ultrasound time	12.96	1	12.96	334.26	<0.0001	
B-Ultrasound temperature	19.87	1	19.87	512.57	<0.0001	
C-enzymatic hydrolysis pH	3.05	1	3.05	78.76	<0.0001	
AB	0.6650	1	0.6650	17.15	0.0043	
AC	0.7329	1	0.7329	18.91	0.0034	
BC	2.39	1	2.39	61.65	0.0001	
A^2^	35.35	1	35.35	911.86	<0.0001	
B^2^	33.52	1	33.52	864.62	<0.0001	
C^2^	70.28	1	70.28	1813.08	<0.0001	
Residual	0.2713	7	0.0388			
Lack of Fit	0.1195	3	0.0398	1.05	0.4627	not significant
Pure Error	0.1519	4	0.0380			
Cor Total	194.74	16				

**Table 3:** Reliability analysis of regression model.

Source		Source	
Std. Dev.	0.1969	R^2^	0.9986
Mean	114.95	Adjusted R^2^	0.9968
C.V. %	11.32	Predicted R^2^	0.9890
		Adeq Precision	665.3862

**Table 4:** Verification of response surface prediction results.

	Ultrasound Time (Min)	Ultrasound Temperature (°C)	Enzymatic Hydrolysis pH	Yield (%)
Predicted value	23.455	84.475	5.012	19.872
Actual value	24.0	85.0	5.0	19.74 ± 0.51 *p* > 0.05

**Table 5:** Yield and chemical composition of TMP-UAE and TMP-HW.

	Yield (%)	Sugar Content (%)	Protein Content (%)
TMP-HW	4.63	37.39	9.17
TMP-UAE	19.74	65.02	9.79

**Table 6:** Lifespan analysis of NL5901 *C. elegans*.

Group	Mean Survival Day ± SEM	Percent Increase
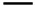 Control	7.940 ± 0.50	
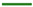 TMP	10.70 ± 0.77	34.76%

Note: The parameters were calculated from survival curves. The data are presented by the mean ± SEM from three independent experiments. All experiments were performed under the same conditions.

## Data Availability

The original contributions presented in the study are included in the article/Supplementary Materials, further inquiries can be directed to the corresponding author.

## Supplementary Materials

The following supporting information can be downloaded at: https://www.mdpi.com/article/10.3390/foods13244150/s1, Figure S1: Effects of Cellulase addition amount; Table S1: Box–Behnken response surface design and corresponding response values of combined enzyme addition; Table S2: Reliability analysis of regression model; Table S3: Regression model analysis of variance; Table S4: Verification of response surface prediction results.
