# Supplementary material for: Ultrasound-Assisted Enzymatic Extraction of Polysaccharides from Tricholoma matsutake: Optimization, Structural Characterization, and Inhibition of α-Synuclein Aggregation
Source: Foods. 2024 Dec 21;13(24):4150. doi: 10.3390/foods13244150 (PMC11675543; doi:10.3390/foods13244150)
Supplement: Supplementary file 1 [file foods-13-04150-s001.zip › foods-3326244-supplementary/foods-3326244-supplementary.pdf]

# Ultrasound-assisted enzymatic extraction of polysaccharides from *Tricholoma matsutake*: optimization, structural characterization and inhibition of $\alpha$-synuclein aggregation

Wen Gao, Yang Wang, Fuping Lu, Fufeng Liu*

Key Laboratory of Industrial Fermentation Microbiology, Ministry of Education, Tianjin, 300457, P. R. China; Tianjin Key Laboratory of Industrial Microbiology, Tianjin, 300457, P. R. China; College of Biotechnology, Tianjin University of Science and Technology, Tianjin 300457, P. R. China;

**Corresponding Author**

*Fufeng Liu, Phone: +86-022-60602717; Fax: +86-022-60602298; E-mail: fufengliu@tust.edu.cn

## Supplementary Tables

Table S1. Box-Behnken response surface design and corresponding response values of combined enzyme addition

| Run | A: Cellulase addition amount (%) | B: Pectinase addition amount (%) | C: Dispase addition amount (%) | Yield (%) | |
|---|---|---|---|---|---|
| | | | | Actual | Predicted |
| 1 | 1 | 0.6 | 1 | 10.98 | 10.96 |
| 2 | 1 | 0.6 | 1 | 10.95 | 10.96 |
| 3 | 1.2 | 0.6 | 1.2 | 10.28 | 10.25 |
| 4 | 1 | 0.6 | 1 | 11 | 10.96 |
| 5 | 1 | 0.8 | 1.2 | 10.31 | 10.32 |
| 6 | 1.2 | 0.8 | 1 | 10.84 | 10.87 |
| 7 | 1 | 0.6 | 1 | 10.87 | 10.96 |
| 8 | 1 | 0.4 | 0.8 | 9.69 | 9.68 |
| 9 | 1.2 | 0.4 | 1 | 10.36 | 10.39 |
| 10 | 1.2 | 0.6 | 0.8 | 10.51 | 10.49 |
| 11 | 1 | 0.8 | 0.8 | 10.3 | 10.29 |
| 12 | 1 | 0.4 | 1.2 | 9.27 | 9.28 |
| 13 | 1 | 0.6 | 1 | 11 | 10.96 |
| 14 | 0.8 | 0.4 | 1 | 9.071 | 9.05 |
| 15 | 0.8 | 0.6 | 1.2 | 9.29 | 9.31 |

| | | | | | |
|---|---|---|---|---|---|
| 16 | 0.8 | 0.6 | 0.8 | 9.41 | 9.44 |
| 17 | 0.8 | 0.8 | 1 | 10.25 | 10.22 |

Table S2. Reliability analysis of regression model

| Source | | Source | |
|---|---|---|---|
| Std. Dev. | 0.0505 | $R^2$ | 0.9975 |
| Mean | 10.26 | Adjusted $R^2$ | 0.9944 |
| C.V. % | 0.4919 | Predicted $R^2$ | 0.9842 |
| | | Adeq Precision | 49.46 |

Table S3. Regression model analysis of variance

| Source | Sum of Squares | df | Mean Square | F-value | P-value | |
|---|---|---|---|---|---|---|
| Model | 7.24 | 9 | 0.8044 | 315.88 | < 0.0001 | significant |
| A-Cellulase addition amount | 1.97 | 1 | 1.97 | 773.28 | < 0.0001 | |
| B-Pectinase addition amount | 1.37 | 1 | 1.37 | 537.48 | < 0.0001 | |
| C-Dispase addition amount | 0.0722 | 1 | 0.0722 | 28.35 | 0.0011 | |
| AB | 0.1222 | 1 | 0.1222 | 47.97 | 0.0002 | |
| AC | 0.0030 | 1 | 0.0030 | 1.19 | 0.3118 | |
| BC | 0.0462 | 1 | 0.0462 | 18.15 | 0.0037 | |
| $A^2$ | 0.7601 | 1 | 0.7601 | 298.48 | < 0.0001 | |
| $B^2$ | 0.6902 | 1 | 0.6902 | 271.04 | < 0.0001 | |
| $C^2$ | 1.85 | 1 | 1.85 | 726.00 | < 0.0001 | |
| Residual | 0.0178 | 7 | 0.0025 | | | |

| | | | | | | |
|---|---|---|---|---|---|---|
| Lack of Fit | 0.0060 | 3 | 0.0020 | 0.6808 | 0.6083 | not significant |
| Pure Error | 0.0118 | 4 | 0.0030 | | | |
| Cor Total | 7.26 | 16 | | | | |

Table S4. Verification of response surface prediction results

| | Cellulase addition amount (%) | Pectinase addition amount (%) | Dispase addition amount (%) | Yield (%) |
|---|---|---|---|---|
| Predicted value | 1.14 | 0.57 | 0.94 | 11.03 |
| Actual value | 1.15 | 0.60 | 0.95 | 10.95 ± 0.31 |

## Supplementary Figure

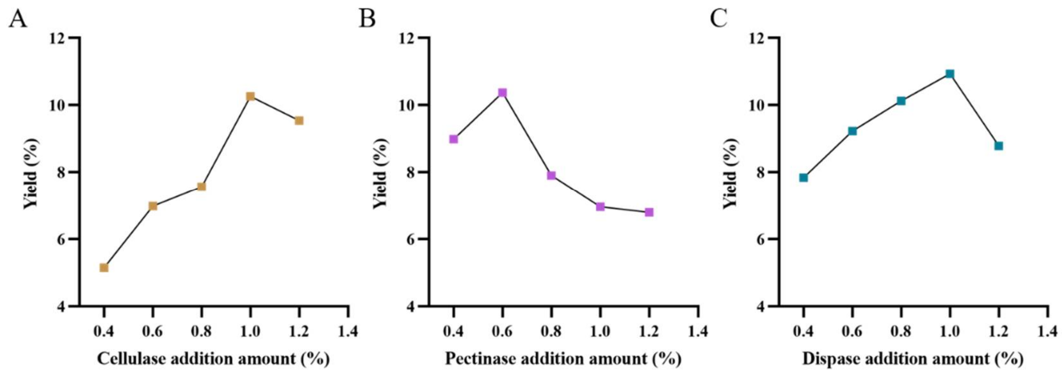


**Figure S1.** Effects of Cellulase addition amount(A), Pectinase addition amount(B) and Dispase addition amount(C) on the yield of TMP.
